# Low-angle subduction of the Indian plate and megathrust geometry below the Eastern Himalayas

**DOI:** 10.1093/nsr/nwaf460

**Published:** 2025-10-28

**Authors:** Ling Bai, Hongru Li, Zhiwen Chen, Huili Zhan, Guohui Li, James Mori, Lin Ding

**Affiliations:** State Key Laboratory of Tibetan Plateau Earth System, Environment and Resources (TPESER), Institute of Tibetan Plateau Research, Chinese Academy of Sciences, Beijing 100101, China; University of Chinese Academy of Sciences, Beijing 100049, China; State Key Laboratory of Tibetan Plateau Earth System, Environment and Resources (TPESER), Institute of Tibetan Plateau Research, Chinese Academy of Sciences, Beijing 100101, China; University of Chinese Academy of Sciences, Beijing 100049, China; State Key Laboratory of Tibetan Plateau Earth System, Environment and Resources (TPESER), Institute of Tibetan Plateau Research, Chinese Academy of Sciences, Beijing 100101, China; University of Chinese Academy of Sciences, Beijing 100049, China; State Key Laboratory of Tibetan Plateau Earth System, Environment and Resources (TPESER), Institute of Tibetan Plateau Research, Chinese Academy of Sciences, Beijing 100101, China; University of Chinese Academy of Sciences, Beijing 100049, China; Key Laboratory of Earthquake Prediction, Institute of Earthquake Forecasting, China Earthquake Administration, Beijing 100036, China; Disaster Prevention Research Institute, Kyoto University, Kyoto 611-0011, Japan; State Key Laboratory of Tibetan Plateau Earth System, Environment and Resources (TPESER), Institute of Tibetan Plateau Research, Chinese Academy of Sciences, Beijing 100101, China; University of Chinese Academy of Sciences, Beijing 100049, China

**Keywords:** continental plate subduction, megathrust geometry, earthquake source parameters, crustal stress field, Eastern Himalayas

## Abstract

The Eastern Himalayas region represents active intercontinental convergence and tectonic strike-slip extrusion. It frequently produces large earthquakes that devastate population centres for millions of people. However, there is no accepted explanation for how the widespread strike-slip motion and plate convergence deformation cause the frequent earthquakes. Here, we use new data from our recently deployed broadband seismic array in the Eastern Himalayas region to determine the regional stress field and detailed structural information about the converging plates. The stress field obtained from the earthquake focal mechanisms shows dominant north–south horizontal compression. Across the Eastern Himalayas region from south to north, the identified Indian crust exhibits low-angle subduction of the crust–mantle boundary Moho and flat-ramp geometry of the plate interface termed as the Main Himalayan Thrust. We suggest that the generation of megathrust earthquakes and the uplift of the broad mountains beneath the Eastern Himalayas region can be explained by the dominant north–south compression and a gentle underthrusting of the Indian plate.

## Teaser

The generation of megathrust earthquakes and the uplift of extensive mountain ranges at the eastern end of the Himalayas can be explained by the dominant north-south compression and a gentle underthrusting of the Indian plate.

## INTRODUCTION

**Figure 1.**
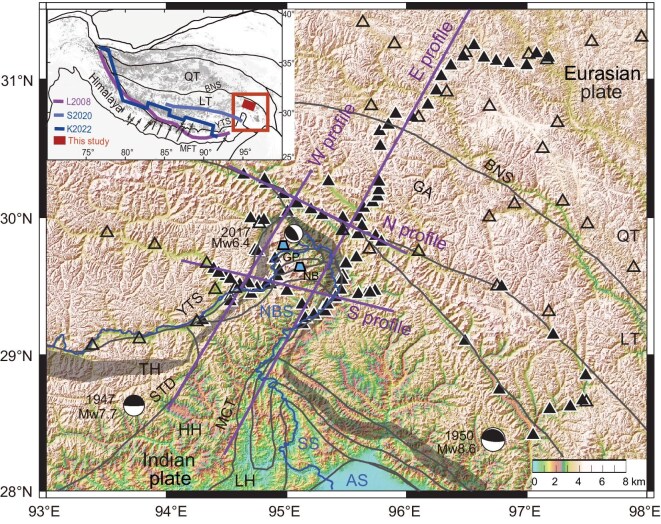
Tectonic map with seismic stations. The solid triangles are our seismic stations (Table S1) and the open triangles are seismic stations from previous studies (see ‘Materials and methods’). The purple lines indicate the locations of the cross sections shown in Figs 3 and 4. The gray area represents the Yarlung Tsangpo Suture, which separates the Higher Himalaya from the TP. The black curves are sutures and active faults [8]. The blue curve is the Yarlung Tsangpo river. The inset in the upper left corner shows the study area in the TP (red rectangle) and constraints on Indian crustal front from receiver functions: S2020 [15]; tomographic images: L2008 [17]; and geochemical isotopes: K2022 [20]. The arrows indicate the crustal deformation constrained by Global Positioning System measurement [11–13]. BNS, Bangong Nujiang Suture; YTS, Yarlung Tsangpo Suture; STD, South Tibetan Detachment; MCT, Main Central Thrust; MBT, Main Boundary Thrust; NBS, Namche Barwa Syntaxis; SS, Siang Syntaxis; AS, Assam Syntaxis; LT, Lhasa Terrane; QT, Qiangtang Terrane; HH, Higher Himalaya; LH, Lesser Himalaya; NB, Namche Barwa metamorphic massif; GP, Gyala Peri metamorphic massif; GA, Gangdese arc.

The Indian and Eurasian plates converge along the Himalayas, causing widespread crustal shortening and large earthquakes in and around the Tibetan Plateau (TP) [1–3]. Understanding the active evolution of the plate boundary process is key to unraveling the mechanism of Himalayan growth [4,5] and the generation of megathrust earthquakes [6,7]. In the Eastern Himalayas region, the orientation of the plate boundary rotates rapidly from east–west along the Himalayas to north–south along the Indo-Burma arc, indicating clear structural variations in the region [8–10]. In addition, the internal shortening rates along the Himalayan front increase markedly from west to east [11–13], suggesting a shorter earthquake recurrence interval in the Eastern Himalayas region (Fig. 1, inset). Our seismic observations at the Eastern Himalayas region provide new constraints on the crustal stress field and the megathrust geometry for this remote region of the continental collision zone.

The downgoing Indian plate exhibits lateral variations both along and across the strike of the Himalayas [14,15]. In the western part of the Himalayas, the Indian Moho extends northwards around the Yarlung Tsangpo Suture (YTS) with a consistently gentle dip angle (7° on average) [15,16]. A west–east transition model from shallow-dipping to steep-dipping subduction has been proposed along the strike of the Himalayas [17–20] (Fig. 1, inset). Further to the southeast, there is a deep continental subduction zone beneath the Burma arc with an average dip angle of >20° [21]. These observations suggest a steeper Indian Moho in the Eastern Himalayas region. However, crustal structures derived from geophysical and geological studies reveal a relatively flat Moho and a rather complex lower crust beneath the eastern end of the Himalayas [10,22]. Lateral variations in the Moho boundary and in the megathrust geometry play important roles in the rupture behavior of large earthquakes [7,23] and in the evolution process of the TP [4,5].

In addition to the continental collision, large-scale tectonic extrusion occurs in the Eastern Himalayas region [24–27]. The 1950 M_W_ 8.6 Assam earthquake and the 1947 M_W_ 7.7 Lang County earthquake are representative of earthquakes that have occurred in the tectonic setting of the compressional and strike-slip stresses [28–30]. Below the Eastern Himalayas region, the YTS forms a south-facing U-shaped shear zone, separating the Tibetan lithosphere from the Himalayan mountains [8–10]. Due to the active mountain building, earthquake fault-plane solutions are diverse [6,10,29]. Widespread strike-slip motions occur in the context of the dominant plate convergence deformation. The detailed crustal structure and stress field in the Eastern Himalayas region is uncertain due to the local spatial heterogeneity and harsh environmental conditions that limit field observations.

Since 2015, we have deployed 90 broadband seismic stations in and around the Eastern Himalayas region (Fig. 1, Fig. S1 and Table S1). This is the first dense broadband seismic network for this remote region. A linear array extends northeastwards across the Himalayas and much of the eastern TP. Our network of stations to the west and east of the array also provides data. We present new results for earthquake sources and stress fields for the entire study area. We also present receiver function images for four profiles: two oriented generally perpendicular (N30°E) to the main trend of the Eastern Himalayas region (the eastern long profile traversing the Lesser Himalaya (LH), the Higher Himalaya (HH), the Lhasa Terrane (LT) and the Qiangtang Terrane (QT)) and two oriented roughly parallel (E20–30°S) to the Himalayan trend (traversing the central and northern parts of the HH). In addition, there were 31 temporary broadband seismic stations deployed between 2003 and 2004 in the northern part of the study area [10,31]. The network and linear array seismic stations provide constraints on the earthquake locations, fault-plane solutions and the continental collision geometry of the downgoing Indian plate (see ‘Materials and methods’).

## RESULTS

### Earthquake focal mechanisms and north–south compression

**Figure 2.**
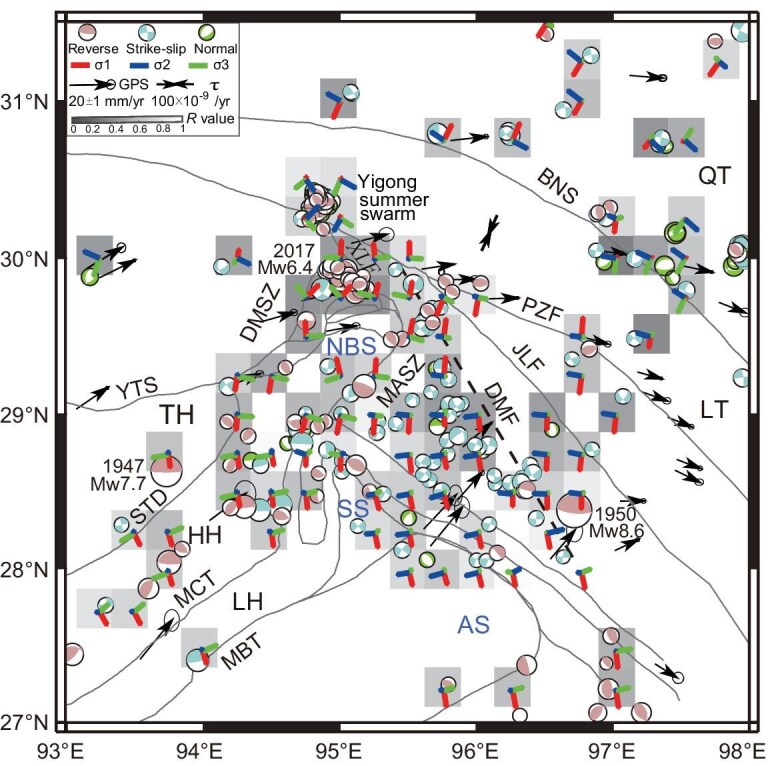
Earthquake relocations, focal mechanisms and the optimal solutions of the compressional stress fields. Beach balls represent thrust, strike-slip and normal fault focal mechanisms. Short bars indicate maximum (${\sigma }_1$), intermediate (${\sigma }_2$) and minimum (${{\mathrm{\sigma }}}_3$) compressional stress directions, with longer lengths for larger plunge angles. The arrows and the vector pair indicate the crustal deformation and horizontal principal strain rates (*τ*), as constrained by the Global Positioning System measurements [11–13]. The gray rectangles represent the relative stress magnitude (*R*) for each grid. The dotted line indicates the inferred Damu fault, which marks the eastern end of the densely distributed strike-slip earthquakes. DMSZ, Dongjiu–Mainling shear zone; MASZ, Medog–Aniqiao shear zone; JLF, Jiali fault; PZF, Parlungzangbu fault. Other symbols are the same as in Fig. 1.

The earthquake activity of the region reflects the deformation pattern and the driving stress field for the Himalayas. We pick seismic wave arrival times to determine relocations [32] and use waveform modeling to calculate the focal mechanisms [33] of 164 M_W_ ≥ 3.0 earthquakes that occurred between 2003 and 2022 (Fig. S1 and Table S2). After the initial continental collision at ∼50 Ma in the Eastern Himalayas region [5], three tectonic units were formed from north to south: the antiformal Namche Barwa Syntaxis (NBS), the synclinal valley of the Siang Syntaxis (SS) and the sedimentary basin of the Assam Syntaxis (AS) [8,9]. Thrust fault earthquakes are dominant in the frontal part of the collisional units, i.e. the northeastern NBS, the western SS and the eastern AS. The 2017 M_W_ 6.4 Mailing earthquake, the 1947 M_W_ 7.7 Lang County earthquake and the 1950 M_W_ 8.6 Assam earthquake are located close to the frontal parts of the NBS, the SS and the AS [28–30,34]. Strike-slip earthquakes are widely distributed throughout the study area, especially to the east of the NBS and the SS. The eastern region of these strike-slip earthquakes shows a linear trend suggesting a blind fault, which extends from the northern NBS in the northwest, through Damu Village in the centre to the eastern AS in the southeast (∼N150°E, marked as the Damu fault by the dotted line in Fig. 2).

We use the earthquake focal mechanisms to calculate the stress field (see ‘Materials and methods’), which determines the unified crustal deformation for an active seismogenic environment (Fig. 2 and Fig. S2) [35]. The dataset includes 298 earthquake focal mechanisms, of which 164 are determined in this study (Table S2) and 134 are collected from previous studies (see ‘Materials and methods’). As expected, the dominant feature of the stress map is a maximum horizontal compression oriented in a north–south direction, nearly perpendicular to the main strike of the Himalayas. These maximum principal stresses have large plunge angles throughout the study area (short bars in Fig. 2), indicating an obvious thrust regime. Geodetic observations support that the southeastern TP rotates clockwise around the Eastern Himalayas region, the north–south shortening is dominant and the horizontal strain rate is the largest over the Himalayas [11–13] (Fig. 2). In the frontal part of the collisional units, the earthquakes are predominantly of the thrust type. Although the stress directions in the northwestern NBS are complicated (Fig. S2), the relative stress magnitudes tend toward 0 and the stress regime is close to pure thrust (Fig. 2).

The compression stress can generate earthquakes with both thrust and strike-slip faults, depending on the style of the stress tensor [35,36]. East of the NBS and SS, the active strike-slip earthquakes are also consistent with the regional north–south maximum horizontal compression. In contrast, the minimum principal stresses have small plunge angles within the NBS and SS but larger ones to the east (short bars in Fig. 2). These observations suggest a high degree of extensional attributes in stress types to the east of the NBS and SS. The combined effects of maximum horizontal compression in the north–south direction and minimum horizontal compression in the east–west direction constitute the stress background for strike-slip earthquakes.

The Cenozoic convergence between the Indian and Eurasian plates resulted in large-scale crustal shortening across the TP [36]. Throughout the study area, the stress field is consistently in a north–south orientation, representing the cumulative effect of long-term stress due to the northward advance of the Indian plate. North–south compression is dominant throughout the region for both the thrust earthquakes in the west and the strike-slip earthquakes in the east of the study area. Such compression explains the folds of the NBS, SS and AS that trend from north to south. The uplift of the HH and the formation of the Namche Barwa and Gyala Peri metamorphic massifs are the most dramatic examples of the north–south compressional tectonics [37].

### North–south profiles from the LH to the QT

**Figure 3.**
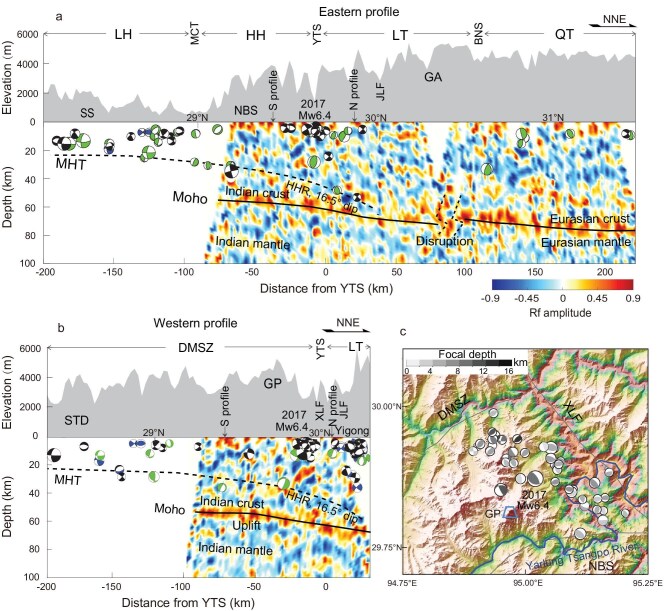
Receiver function images with earthquake relocations and focal mechanisms for the north–south profiles. (a) The eastern profile and (b) the western profile. The profiles are shown in Fig. 1. The solid lines are the inferred Moho and the dotted lines are the inferred MHT. HHR indicates the Higher Himalayan ramp of the MHT with a dip angle of 16.5°. There is no vertical exaggeration in these figures. (c) The 2017 Mw 6.4 Mailing earthquake and its aftershocks. Other symbols are the same as in Figs 1 and 2.

We use receiver function analyses to clarify the details of the physical structure of the convergent plates (Fig. 3 and Fig. S3; see ‘Materials and methods’) [38,39]. Figure 3a shows a 400-km-long seismic profile comprising 300 km for the receiver function image and 100 km for the earthquake distribution. The profile extends from SSW to NNE, and traverses the LH, the HH, the LT and the southern QT. The strong positive phase at depths of 55–75 km is the P-to-S converted phase at the Moho. This feature is consistent throughout the profile and is indicated by the solid line labeled Moho. From the NBS to the YTS, the Moho gradually increases from 55 to 60 km with a shallow dip angle of ∼5°. From the YTS to the Jiali strike-slip fault, the dip angle of the Moho increases from 60 to 70 km with a dip angle of ∼10°. North of the Jiali fault are the Gangdese Mountains—the magmatic arc that was formed by the subduction of the Neo-Tethyan oceanic lithosphere and the subsequent continental collision [40,41]. Between the Jiali fault and the Gangdese arc, the Moho gradually increases from 70 to 75 km with a shallow dip angle of ∼5°. Beneath the Gangdese arc at ∼30.5°N, the Moho is vertically disrupted for almost 10 km. Further to the north, the Moho gradually increases from 65 to 75 km and becomes almost flat beneath the QT.

We interpret the Moho disruption beneath the Gangdese arc as the Moho transition between the Indian and Eurasian plates (Fig. 3a). This Moho disruption has also been observed in receiver function images using teleseismic P waves arriving from different directions. When the P waves arrive from the north, the Moho image is sharp in the south, but incoherent north of 30.5°N (Fig. S4A). In contrast, when the P waves arrive from the south, the Moho image is weak in the south, but clearly visible north of 30.5°N (Fig. S4B). These observations provide constraints on the dipping trend of the Moho [14]. In addition, the Poisson’s ratio and seismic anisotropy also differ from south to north around the Gangdese arc. The average Poisson’s ratio obtained from our receiver function analysis is 0.20 in the south, but is 0.27 in the north due to the presence of the quartz-rich granites and metamorphic rocks beneath the Gangdese arc [40,41] (Fig. S5). The fast polarization direction of the upper-mantle seismic anisotropy is NE in the south, but rotated to be SE in the north, suggesting the dominant contribution of the shear wave velocity from the southeastward extrusion of the TP lithosphere beneath the QT [24,42]. These observations indicate that the Moho transits between the Indian and Eurasian plates beneath the Gangdese arc.

In Fig. 3a, the relocated earthquakes show a NNE dipping structure from the LH to the southern LT around the Jiali fault. The focal depths of the earthquakes are constrained well by our local data with good azimuthal coverage (Fig. 1). Beneath the LH, earthquakes are numerous and concentrated at depths of <25 km. Further north, beneath the southern LT, the earthquakes occur at depths of ∼50 km (nos. 36 and 126 in Table S2). The deeper edge of the earthquake corresponds to the dipping trend of the negative signal (blue) of the observed receiver function images. The combination of the earthquake relocations and the receiver function images delineates a NNE dipping structure at depths of 25–55 km. We explain this structure as the Main Himalayan Thrust (the MHT, marked by the dotted line in Fig. 3a). The MHT has been clearly identified for the Central and Western Himalayas, as revealed by earthquake distributions [7,43,44], wave-speed changes [14,16], anisotropic shearing [45,46] or tectonic reconstruction [47,48]. This is the first inferred imaging of the MHT for the Himalayas east of 94°E, where the MHT appears to have less seismic contrast than in the Western and Central Himalayas. The MHT is not a clear signal as the Moho, but is seen intermittently across the profiles. Beneath the HH and the southern LT, the north-dipping MHT formed a ramp with a dip angle of ∼16.5°, which is 10° steeper than the average dip angle of the underlying Moho. These observations suggest that the downgoing Indian crust thins to the north and pinches out to the north of the YTS, consistently with the northern boundary of the active seismic zone.

The western profile (Fig. 3b) follows the western part of the south-facing U-shaped YTS. The Moho depth to the southwest is ∼53 km. To the north, the Moho is slightly elevated beneath the western Gyala Peri metamorphic massif. Further north around the Jiali fault zone, the Moho clearly deepens to ∼68 km. The lower edge of the relocated earthquakes and the negative signal of the receiver function images delineate the NE-dipping structure, marked by the dotted line labeled MHT. The relocated earthquakes above the estimated MHT structure also show increasing depth to the northeast. The 2017 Mw 6.4 Mailing earthquake and its aftershocks occurred at the northeast flank of the NBS, trending northwest and southeast (Fig. 3c). These earthquakes were divided by the Yarlung Tsangpo river into two clusters at the northwest and southeast, respectively. To the northwest, the across-strike distribution of the earthquakes delineates a steeply dipping fault towards the northeast, which is consistent with the north-dipping nodal plane of the Mw 6.4 Mailing earthquake (strike = 328°, dip = 66°, slip = 108°) [34]. Similarly, the along-strike distribution of the earthquakes extends to greater depths toward the southeast, where the foliation of the Dongjiu–Mainling shear zone is subvertical [9,10]. In contrast, to the southeast, earthquakes are dominated by reverse faults and occur at depths of <10 km beneath the surface. The lower depth limit of seismicity shallows to the southeast. These observations suggest that the major tectonic features of the 2017 Mailing earthquake source region are the northeast-dipping thrust combined with the subvertical sharing and the river incision (Fig. S6).

### East–west profiles through the HH

**Figure 4.**
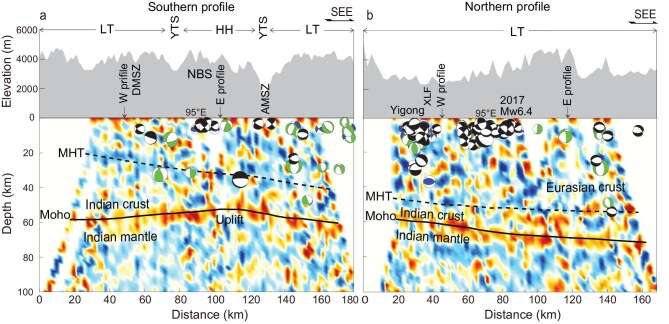
Receiver function images with earthquake relocations and focal mechanisms for the east–west profiles. (a) The southern profile and (b) the northern profile. The locations of the profiles are shown in Fig. 1. The solid lines are the inferred Moho and the dotted lines are the inferred MHT. Other symbols are the same as in Figs 1 and 2.

Figure 4a shows the southern profile that crosses the trend of the NBS as determined by using receiver function analysis. The profile intersects the LT in the west and east, and the HH in the centre. The Moho discontinuity (marked by the solid line labeled Moho) at the eastern and western ends is at a depth of ∼60 km. In contrast, in the central part of the profile, the Moho is elevated to a depth of ∼50 km. Low-velocity anomalies are evident above the Moho, which have been identified by using magnetotelluric and seismic imaging and interpreted as partial melting [49,50]. We outline the MHT based on the earthquake relocations, focal mechanism solutions, the weak east-dipping trend of the low-velocity anomalies and the constraint provided by the intersection of the eastern and western profiles. These observations suggest the presence of the MHT at depths of 20–40 km.

North of the NBS is the Jiali strike-slip fault, which accommodates deformation from the southeastern extrusion of the TP [24,25]. The northern profile follows the central segment of the Jiali fault (Fig. 4b). From northwest to southeast, the depth of the Moho increases from 58 to 72 km. Earthquakes in the southeastern part of the profile are located at depths of ∼50 km (nos. 36 and 126 in Table S2). The estimated MHT in the northern profile is between 45 and 55 km, which is ∼10–20 km deeper than that in the southern profile. The Indian crust in the northern profile is thinner than that in the southern profile, but is still 15–20 km thick. These observations suggest that the Indian crust has thinned and extended northward beyond the YTS into the LT.

## DISCUSSION

### Low-angle subduction of the Indian plate below the Eastern Himalayas region

The Himalayas are the most prominent example of an active collisional orogen on Earth. Various models have been proposed to interpret the evolution of the Himalayan mountains, including thrust duplexing, crustal flow, eclogitization and mantle delamination. The geometry of the downgoing Indian plate is considered the primary constraint on the models [5,16]. The northern limit of the Indian crust beneath the Himalayas varies from west to east and the detailed structure beneath the Eastern Himalayas region is debated. As predicted by the west-to-east transition model of shallowly dipping underplating and steeply dipping subduction [17–19], the Indian plate is expected to steepen across the Eastern Himalayas region. However, these models appear rather simplistic when compared with regional-scale crustal structures from geophysical, geological and geochemical studies. At the western TP at ∼81.5°E, the Indian crustal front is located around the YTS with a crustal thickness of <15 km [16,17,20]. At the central TP at ∼85–87°E, the Indian lower crust extends horizontally beneath the southern LT to a maximum of 31°N [14,17,20,45,51]. At the eastern TP at ∼92–94°E, the Indian lower crust is either restricted to the south of the YTS [17,20] or further extended to the LT [22,52,53]. The interpreted crustal structures of the Eastern Himalayas region are non-unique due to small-scale velocity anomalies [17] and localized continental loss [20]. Slab tearing has been proposed to explain the morphology of the Indian slab for the Eastern Himalayas region [15,52,53] because of the large lateral variations in the Moho depths. These lateral variations in the crustal structure play an important role in the mountain uplift and the evolution of the TP [4,5].

Our eastern broadband seismic profile is located at 94–97°E, spanning from the LH to the QT. Due to the inaccessibility of the Gangdese mountain area, there is a 25-km gap between stations in the LT. For the nearby 10 stations within 100 km across the gap, we obtained 700 high-quality receiver functions, which are sufficient to image the Moho at depths of ∼70 km. Based on our new receiver function images, we found that the downgoing Indian crust deepens from 55 km below the HH to 75 km below the LT, with an average dip angle of ∼7°. Beneath the LT at ∼30.5°N, there is a 10-km vertical Moho disruption, which we interpret as the Moho transition zone between the Indian and Eurasian plates in consideration of Poisson’s ratio, seismic anisotropy and teleseismic P waves arriving from different directions (Fig. S5). These observations suggest that the downgoing Indian plate is underplating beneath the Eastern Himalayas region along a shallow dip plane, rather than being subducted into the mantle at a steep angle. In addition, the Indian crust continues to extend farther beyond the YTS until the Gangdese arc in the LT. Seismic tomography and receiver function images from regional seismic observations support this shallowly dipping Indian plate subduction model for the Eastern Himalayas region [22,52,53].

### Megathrust geometry below the Eastern Himalayas region

Traditional models suggest that the Eastern Himalayas region is a crustal-scale accretionary prism so that horizontal shortening is distributed throughout the prism. Since the 1980s, the MHT has been identified in the Central Himalayas so that major faults are rooted in a common mid-crustal decollement [43]. The MHT has been recognized as being generally responsible for megathrust earthquakes and the Himalayan uplift [7,54,55]. By combining earthquake source parameters with receiver function images, we have newly identified the MHT around the Eastern Himalayas region (Figs 3 and 4). Below the LH, the MHT is almost flat at a depth of 20–25 km. Beneath the HH and the southern LT, the MHT deepens from 25 to 55 km with an average dip angle of 16.5°, defining a Higher Himalayan ramp. The crustal shortening within the Himalayan accretionary wedge driven at the base by various types of dislocation on the MHT contributes to the uplift of the HH, as seen in the Namche Barwa and the Gyala Peri metamorphic massifs.

The geometry of the Himalayas’ megathrust provides clues for understanding the rupture behavior of large earthquakes [56]. Thrust mega-earthquakes are more likely to rupture shallowly dipping faults than steeply dipping faults [23,57]. The 1947 M_W_ 7.7 Lang County earthquake is located close to the southern HH unit, where the MHT geometry is nearly flat and the stress field is compressional. Similarly, the 1950 M_W_ 8.6 Assam earthquake is located close to the LH unit. Although the source area of the 1950 Assam earthquake has both compressional and shearing stress fields, aftershocks are more likely to be concentrated around the AS than along the Jiali strike-slip fault [30]. In addition, moderate earthquakes are identified as localized on the MHT, although their fault-plane solutions are different (Figs 3 and 4). As the Indian plate continues to underplate beneath the LT, the Indian crust can be thinned by the material transfer or magma upwelling to the upper plate [16]. This process creates steeply dipping faults in the Himalayan orogenic wedge. This northward movement of the Indian plate is associated with the development of a megathrust system consisting of both the flat-ramp geometry of the MHT and the steeper dipping faults above the MHT, as revealed by the 2017 Mailing earthquake.

### A speculated continental collision model for the Eastern Himalayas region

**Figure 5.**
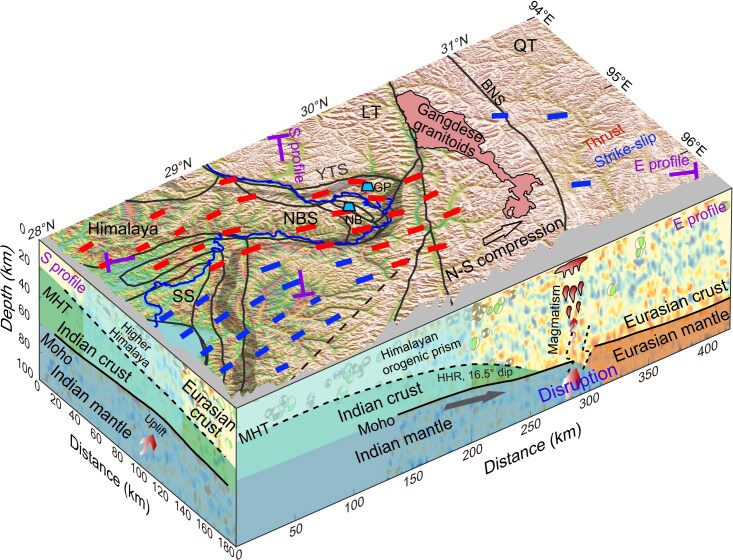
Final model showing crustal structure and stress field around the Eastern Himalayas region. The broad gray feature on the top surface represents the Yarlung Tsangpo Suture (labeled YTS), which separates the NBS from the LT. Two vertical faces are receiver function images along the eastern and southern profiles with earthquakes. Two lines in the surface show the locations of the two profiles. Short bars indicate compressive and shearing stress states. The filled area with curved frame is the Eocene Gangdese granitoids [59]. There is a north–south compressional stress field in the Tibetan crust (open arrow on the surface) and low-angle subduction of the Indian crust (gray arrow on the north–south panel). Other symbols are the same as in Figs 1 and 2.

Figure 5 summarizes our observations in a final model for the Eastern Himalayas region. Stress directions from earthquakes are plotted on the top surface. Despite the complex regional tectonics, the north–south compression of the Indian–Eurasian plate collision is the primary control for the long-term deformation and earthquake generation of the Eastern Himalayas region [58]. The dominant north–south compression produces reverse faulting earthquakes within the HH. Similarly, the north–south trending maximum horizontal compression and the east–west minimum horizontal compression produce strike-slip earthquakes to the east of the HH. In addition, receiver function images and earthquake source parameters are shown on the north–south and east–west panels, with the inferred Moho and MHT structures (solid and dotted black lines) (Fig. 5 and Fig. S7). There is a vertical disruption in the Moho, representing the Moho transition between the Indian and Eurasian plates. The shallow-dipping Indian Moho and the northward thinning of the MHT indicate that the Indian crust is extended beneath the Eastern Himalayas region far beyond the YTS until the Gangdese granitoids beneath the LT [2,59].

In all of the images, the most prominent amplitudes (subhorizontal features with solid lines) are the wave conversions associated with the Moho. While the Moho is clearly visible in all of the profiles, a weaker feature of a negative signal (northeast-dipping features with dotted lines) associated with the MHT can also be seen, but not as clearly. This indicates that, for seismic wave conversions, the Moho discontinuity is stronger than the MHT mid-crustal decollement. These features at the eastern end of the Himalayas are comparable to those in the Central Himalayas [7], but different from the deep continental subduction features seen in the Western Himalayas [60] and in the Burma arc [21]. Due to the harsh natural environment, it is difficult to deploy dense seismic stations in the Eastern Himalayas region. We rely on the joint analysis of local and teleseismic earthquake data from long-term observations to improve the resolution of continental collision zones. The mechanisms of the 1950 M_W_ 8.6 Assam earthquake and the 1947 M_W_ 7.7 Lang County earthquake have been debated [28–30]. Our study suggests the possibility that these earthquakes are large thrust events that may have ruptured the identified megathrust system. Future studies should address the megathrust geometry for the entire Himalayan belt, so that we can better understand the structural controls of the earthquake generation and the plateau uplift in the continental plate collision zone.

## CONCLUSION

The Eastern Himalayas region represents a complicated tectonic background with continental collision of the Indian and Eurasian plates and southeastward extrusion of the TP. How this plate boundary condition affects the growth of the Himalayas and the generation of large earthquakes is unclear. Here, we have provided a new investigation of the crustal structure and earthquake source parameters by using seismic stations that we deployed in the eastern end of the Himalayas. The tectonic stress derived from the earthquake focal mechanisms shows a north–south horizontal compression. This north–south compressional stress gives rise to reverse fault earthquakes concentrated within the HH and strike-slip fault earthquakes to the east of the HH. The entire downgoing Indian crust has an average dip angle of ∼7° and the Moho extends from a depth of 55 km beneath the HH to 75 km beneath the LT. The newly identified MHT has a flat geometry beneath the LH and a steeper dip angle of ∼16.5° beneath the HH, which accommodates megathrust earthquakes along the entire Himalayan orogenic belt. There is a simple compressional stress field but complicated geological structures throughout the Eastern Himalayas region. These observations provide new constraints for understanding the mountain uplift and the megathrust deformation beneath the active collisional orogen.

## MATERIALS AND METHODS

### Experimental design

Since 2015, we have deployed 90 broadband seismic stations around the Eastern Himalayas region (Fig. 1 and Table S1). These stations are equipped with Geolight PCS or Guralp 3ESP seismometers and Edas 24 HR or Reftek 130 recorders with sampling rates of 40–100 Hz. In addition, 5 permanent broadband stations [61] and 31 temporary broadband seismic stations were deployed in the study area from 2003 to 2004 [10,31]. We used 164 local earthquakes of M_W_ ≥ 3.0 and 1189 teleseismic earthquakes of M_W_ ≥ 5.5 to study the earthquake relocations, focal mechanisms, crustal stress fields and receiver function images.

### Local P- and S-wave arrival times for earthquake relocation

Figure S1A shows the arrival time versus epicentral distance for an example earthquake. Within the epicentral distance of 0–0.5°, the differential P- and S-wave arrival times are constant at ∼7 s, indicating that the focal depth is their primary constraint. Based on the pickup of P and S waves from local seismic stations (Fig. S1B), we determined hypocentres for 164 earthquakes with M_W_ ≥ 3.0 by using the HYPOSAT method [32] (Table S2). We used a four-layer velocity model from Crust1.0 [62] for the P wave and an average P and S wave velocity ratio (Vp/Vs) of 1.704 that we observed in this study for the S-wave velocity (Fig. S1B).

### Waveform inversion for focal mechanism determination

We calculated the focal mechanisms by modeling local seismic waveforms using the CAP (Cut and Paste) method for 164 earthquakes [33] (Fig. S1C and Table S2). The frequency range was 0.05–0.2 Hz for body waveforms and 0.05–0.07 Hz for surface waves. The final focal mechanisms were obtained based on the least-squares misfit between observed and synthetic waveforms. The types of seismic mechanisms are classified according to the range of slip angles *λ* on the fault plane: 45° < *λ* < 135° for the reverse fault, –135° < *λ* < −45° for the normal fault and –180° ≤ *λ* ≤ –135°, –45° ≤ *λ* ≤ 45° or 135° ≤ *λ* ≤ 180° for the strike-slip fault.

### Stress field inversion from focal mechanisms

The estimates of the stress orientation provide constraints on crustal deformation and earthquake physics. We define the range of stress based on the source parameters of the 164 earthquakes obtained in this study and 134 earthquakes collected from previous studies [34,63–68] (Fig. 2). We calculated the maximum (${\sigma }_1$), intermediate (${\sigma }_2$) and minimum (${\sigma }_3$) principal compressive stresses and the relative stress magnitude *R* = (${\sigma }_1 - {\sigma }_2$)/(${\sigma }_1 - {\sigma }_3$) by using the MSATSI program in the MATLAB language [35]. We divided the study area into 0.25° × 0.25° grids. Uncertainty was determined by using 1000 bootstrap resamplings and a minimum of one event per node was established (Fig. S2).

### Receiver function analysis

We calculated P‐to-S receiver functions by using frequency‐domain source equalization deconvolution methods with a Gaussian filter of 2.0 and a water level of 0.001 [38,39]. We selected 1189 teleseismic earthquakes with Mw ≥ 5.5 for 10 327 Ps conversion wave receiver functions (Fig. S3). We obtained images by using the common conversion point stacking method with bin sizes of 2° and a Fresnel zone (Fig. S4) [18,38,39]. Receiver functions were migrated from time to depth by tracing the rays for each station through the Crust1.0 model [61]. Figure S5 shows a comparison of Poisson’s ratios between the LT and the QT. Figure S7 shows the receiver function images for the four profiles with comparisons of Moho images from previous studies [22,50,69,70].

## Supplementary Material

nwaf460_Supplemental_File
