# Supplementary material for: Low-angle subduction of the Indian plate and megathrust geometry below the Eastern Himalayas
Source: Natl Sci Rev. 2025 Oct 28;12(12):nwaf460. doi: 10.1093/nsr/nwaf460 (PMC12704103; doi:10.1093/nsr/nwaf460)
Supplement: nwaf460_Supplemental_File [file nwaf460_supplemental_file.docx]

Supplementary Materials for

**Low-angle subduction of the Indian plate and megathrust geometry below the Eastern Himalayas**

**Ling Bai^1,2,*^, Hongru Li^1,2,*^, Zhiwen Chen^1,2^, Huili Zhan^1,2^, Guohui Li^3^, James Mori^4^, Lin Ding^1,2^**

^1^ State Key Laboratory of Tibetan Plateau Earth System, Environment and Resources (TPESER), Institute of Tibetan Plateau Research, Chinese Academy of Sciences, Beijing 100101, China

^2^ University of Chinese Academy of Sciences, Beijing 100049, China

^3^ Key Laboratory of Earthquake Prediction, Institute of Earthquake Forecasting, China Earthquake Administration, Beijing 100036, China

^4^ Disaster Prevention Research Institute, Kyoto University, Uji, Kyoto 611-0011, Japan

*****Corresponding author. E-mails: bailing@itpcas.ac.cn, lihongru@itpcas.ac.cn

**Supplementary Materials for this paper include the following:**

Figures S1 to S7

Tables S1 to S2


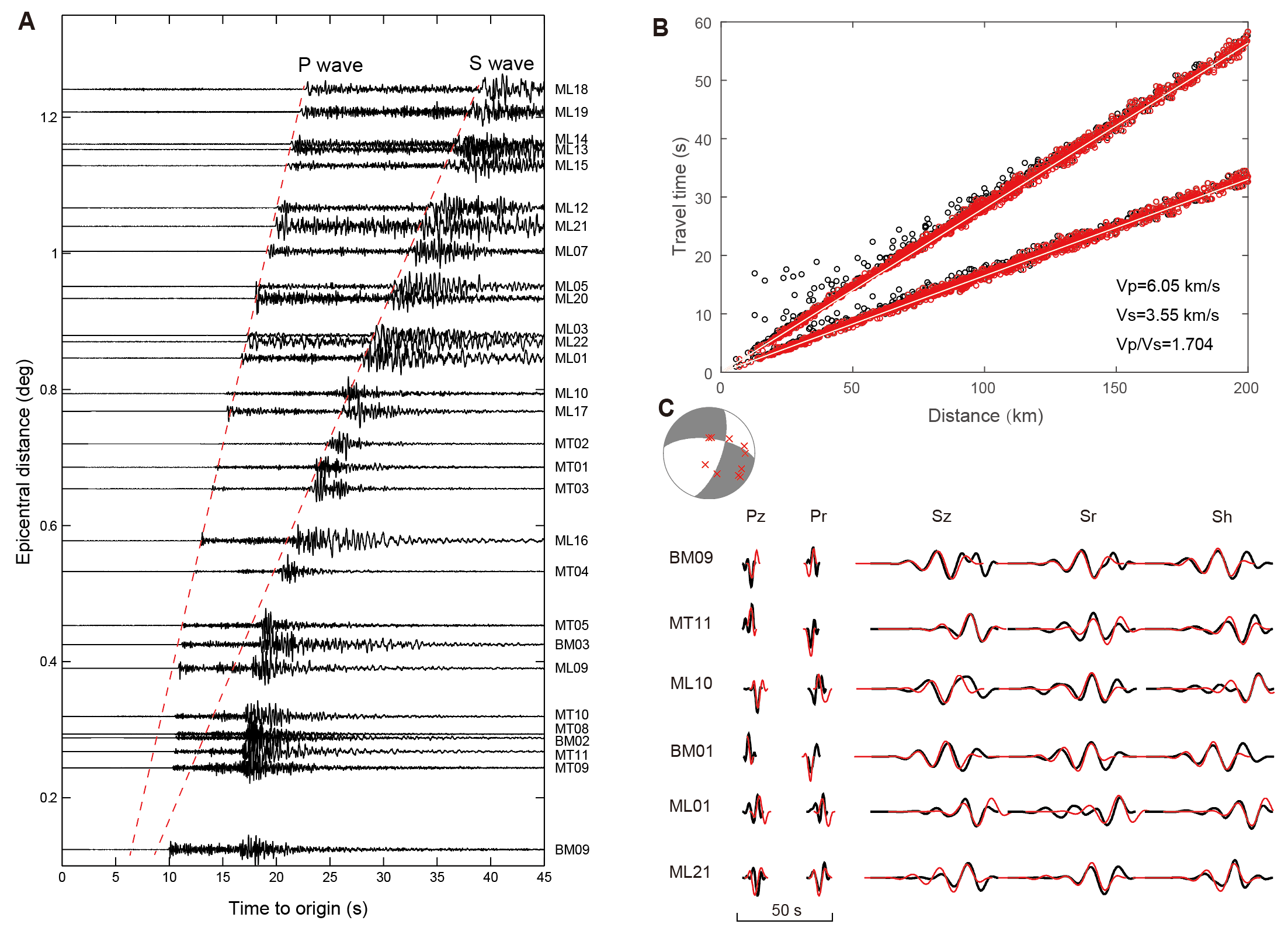


**Fig. S1.** Example data for the earthquake relocation and focal mechanism determination. (A) Record section of vertical component seismograms recorded by our seismic stations (Table S1). The earthquake occurred on December 2, 2020 (No. 126 in Table S2) with a relocated focal depth of 48 km. At smaller distances, the relative arrival times of P- and S-waves remain stable, indicating that the focal depth is their main constraint. The red lines show the predicted P- and S-wave arrival times assuming a shallow earthquake with focal depth near the surface. (B) Hypocentral (red circles) and epicentral (black circles) distances versus travel times for all the 164 earthquakes. The hypocentral distances are calculated using the relocated earthquake focal depths so that the arrival times follow a linear trend. The white lines show a linear fit of the observed arrival times using the gradient parameters listed at the lower-right corner, which represent the average P- and S-wave velocities, and the Vp/Vs ratio of the seismogenic layer. (C) Waveform fitting between observed (black) and theoretical (red) waveforms for the calculation of the focal mechanism of the December 2, 2020 earthquake. The preferred focal mechanism shown in the upper left. The station names are listed to the left of the waveforms.


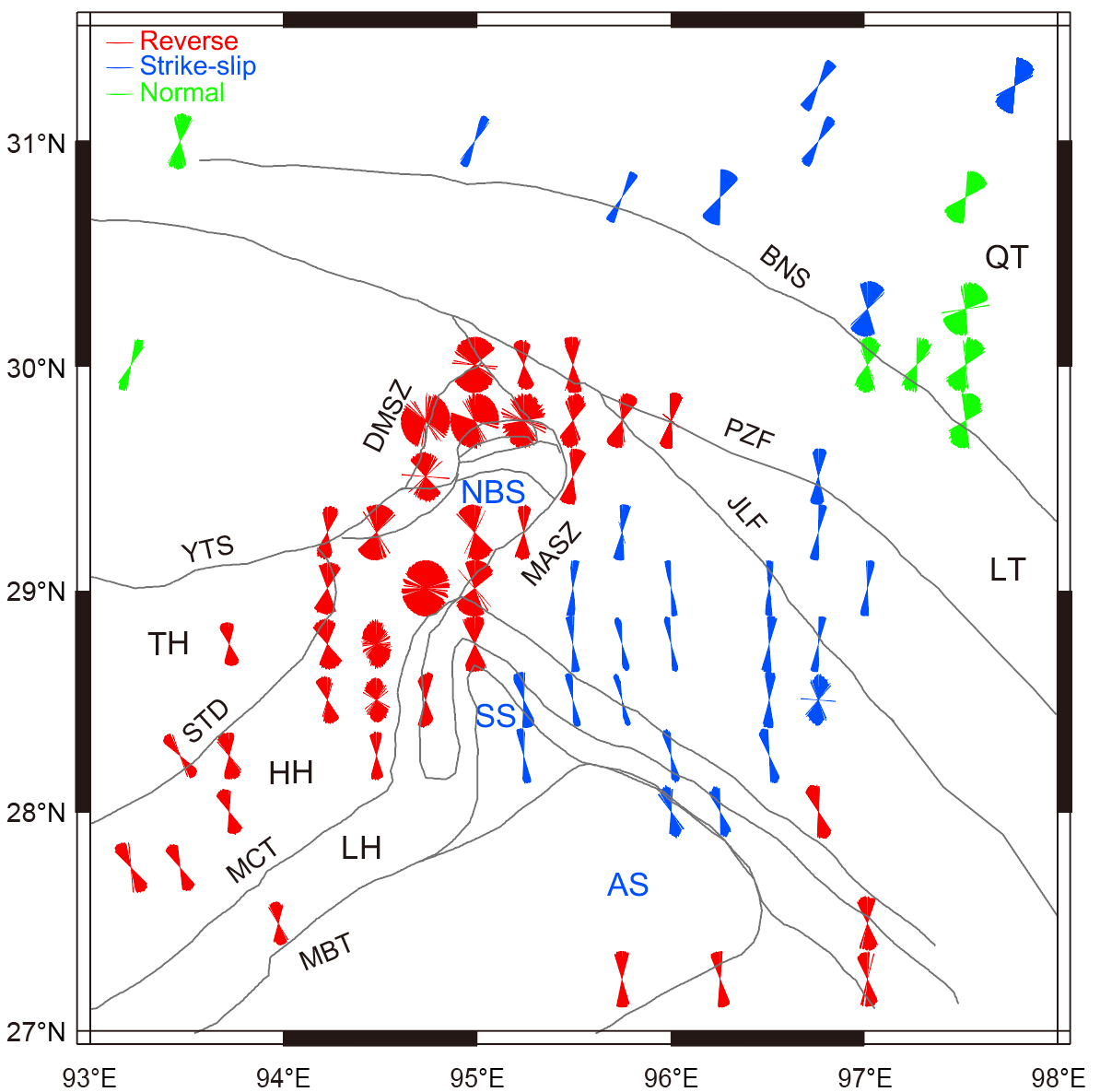


**Fig. S2.**  Distribution of the maximum horizontal stress. Earthquakes occur to the northwest of the NBS (the Yigong summer swarm shown in Fig. 2) are not included in the tectonic stress field calculation (Table S2).


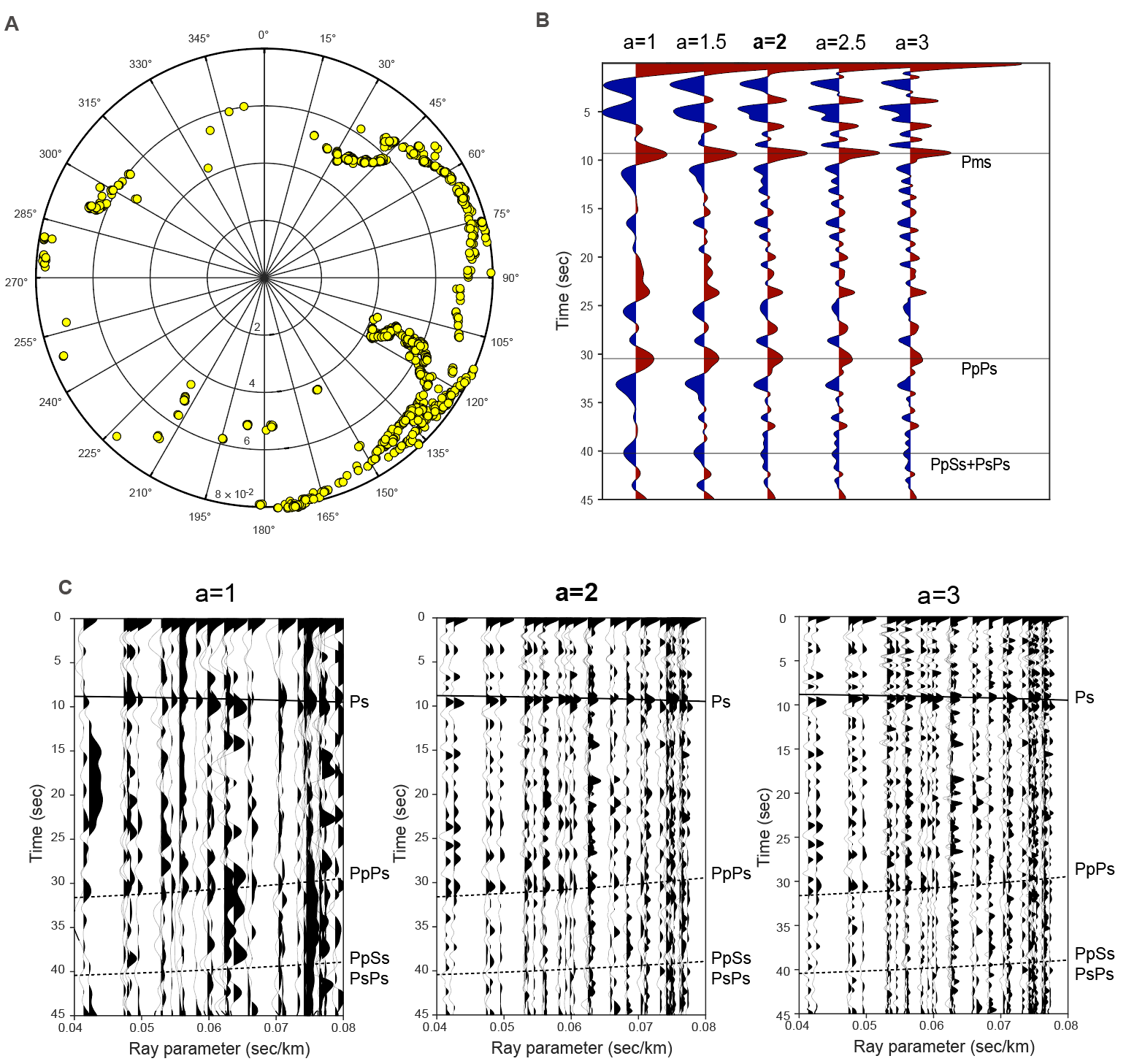


**Fig. S3.** Example data for receiver function analysis. (A) Back azimuth and ray parameter of 1189 teleseismic earthquakes with Mw≥5.5 for Ps conversion wave receiver functions from all seismic stations. Solid dots represent earthquakes. Azimuths range from 0-360° and ray parameters range from 0-0.08. The centre is the average position of our seismic stations. (B) The stacked receiver functions with the Gaussian parameter of a=1, 1.5, 2, 2.5 and 3, respectively. (C) Ray parameter vs. time for the receiver functions with the Gaussian parameter of a=1, 2 and 3, respectively. The waveforms are recorded from the CD05 station that is located in the northeast of the study area.


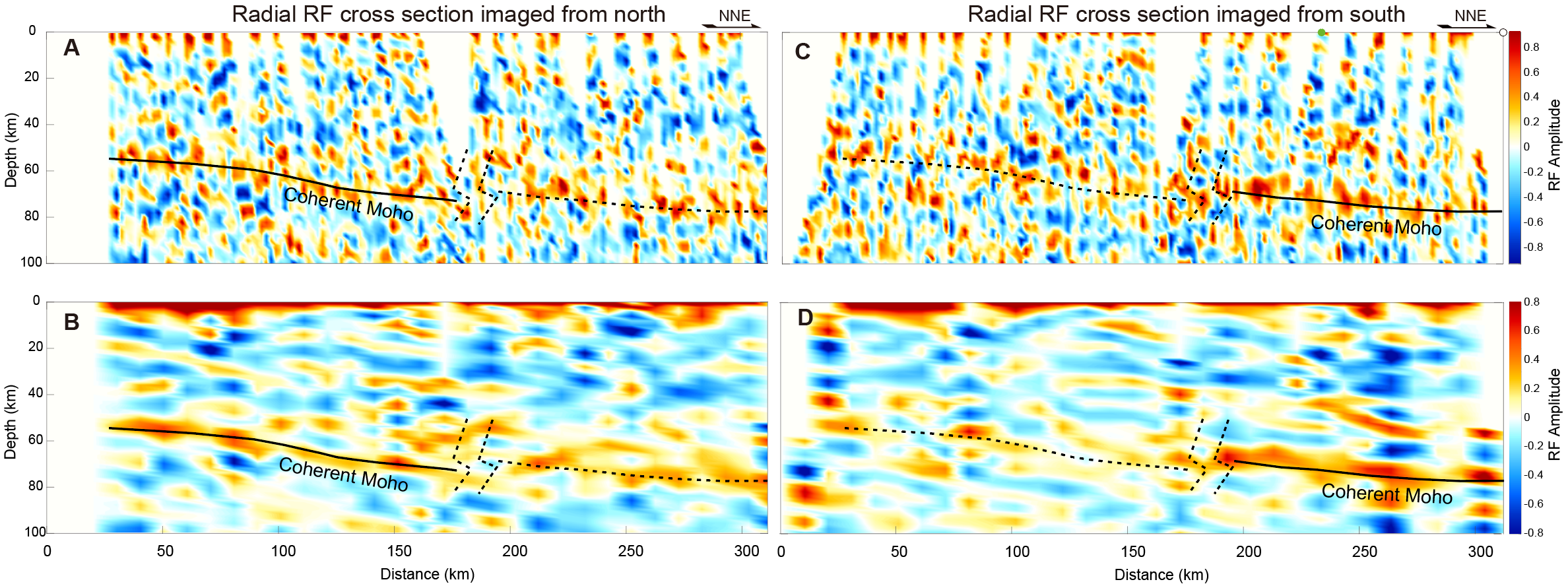


**Fig. S4.** Radial receiver function images along the eastern line using Ps wave ray path from different directions. (A) Receiver function cross section imaged using Ps wave ray path from the north. The Moho image is sharper to the south (in the distance range of 0-150 km) than to the north (in the distance range of 150-300 km). The bin size for common conversion-point stacking is 2°. (B) The same as Fig. S4A but with the bin size of a Fresnel zone. (C) Receiver function cross section imaged using Ps wave ray path from the south. The Moho image is more coherent to the north than to the south. This observation provides constraints on the dipping trend of the Moho. (D) The same as Fig. S4C but with the bin size of a Fresnel zone.


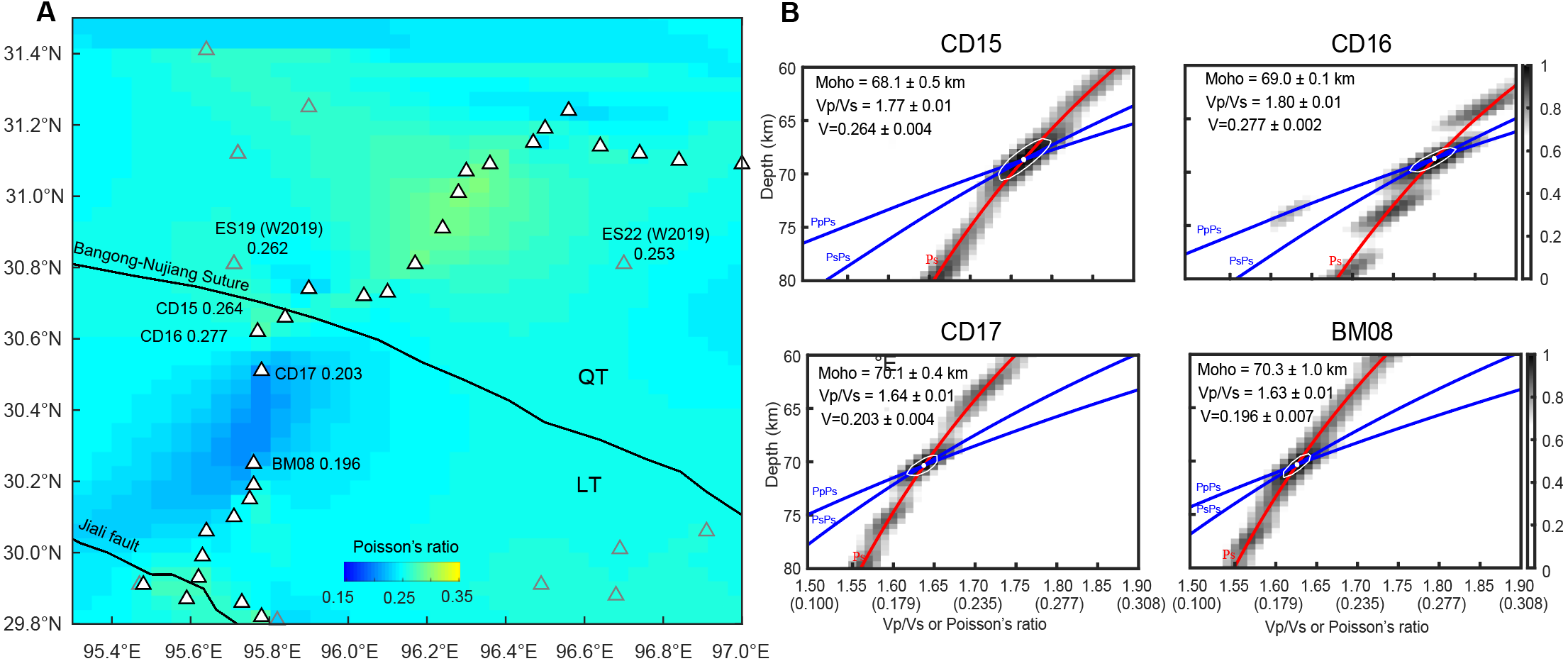


**Fig. S5.** (A) Poisson’s ratio from receiver function analysis. Solid and open triangles are Poisson’s ratio from this study and from the previous study of W2019 [52], respectively. The number next to each station name is the Poisson’s ratio. (B) H-К scan map for four stations around the Moho transition zone. We consider constraints from nearby stations and sensitivity of the back azimuth for the H-К calculations.


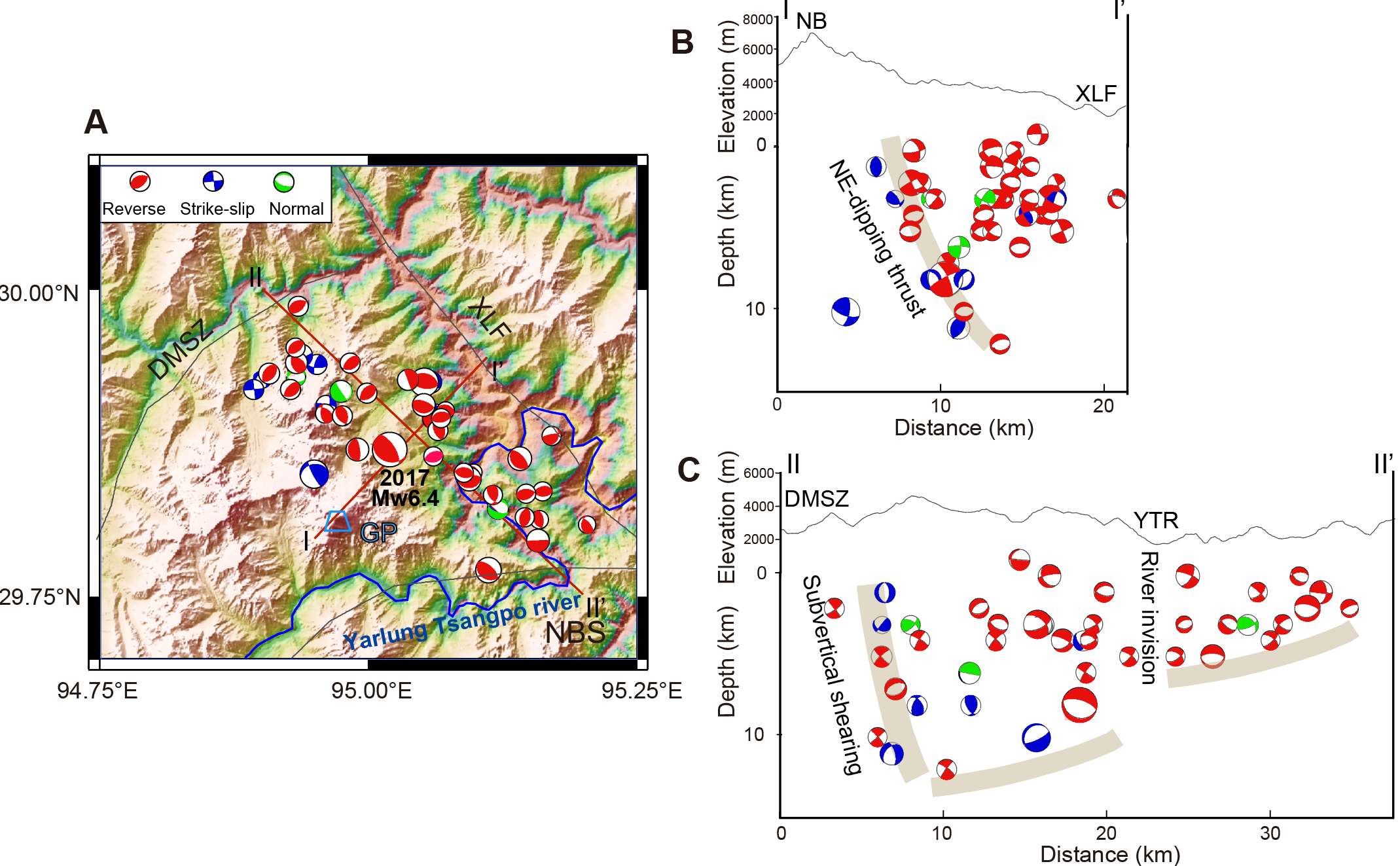


**Fig. S6.** Focal mechanisms and hypocentral relocations of the 2017 Mw6.4 Mainling earthquake and its aftershocks. (A) The map view of the earthquakes. (B) and (C) The profiles along strike (Ⅰ Ⅰ’) and across the strike (II II’) of the aftershock distribution. The major tectonic features of the earthquake source region are the northeast-dipping thrust combined with the subvertical sharing and the river incision.


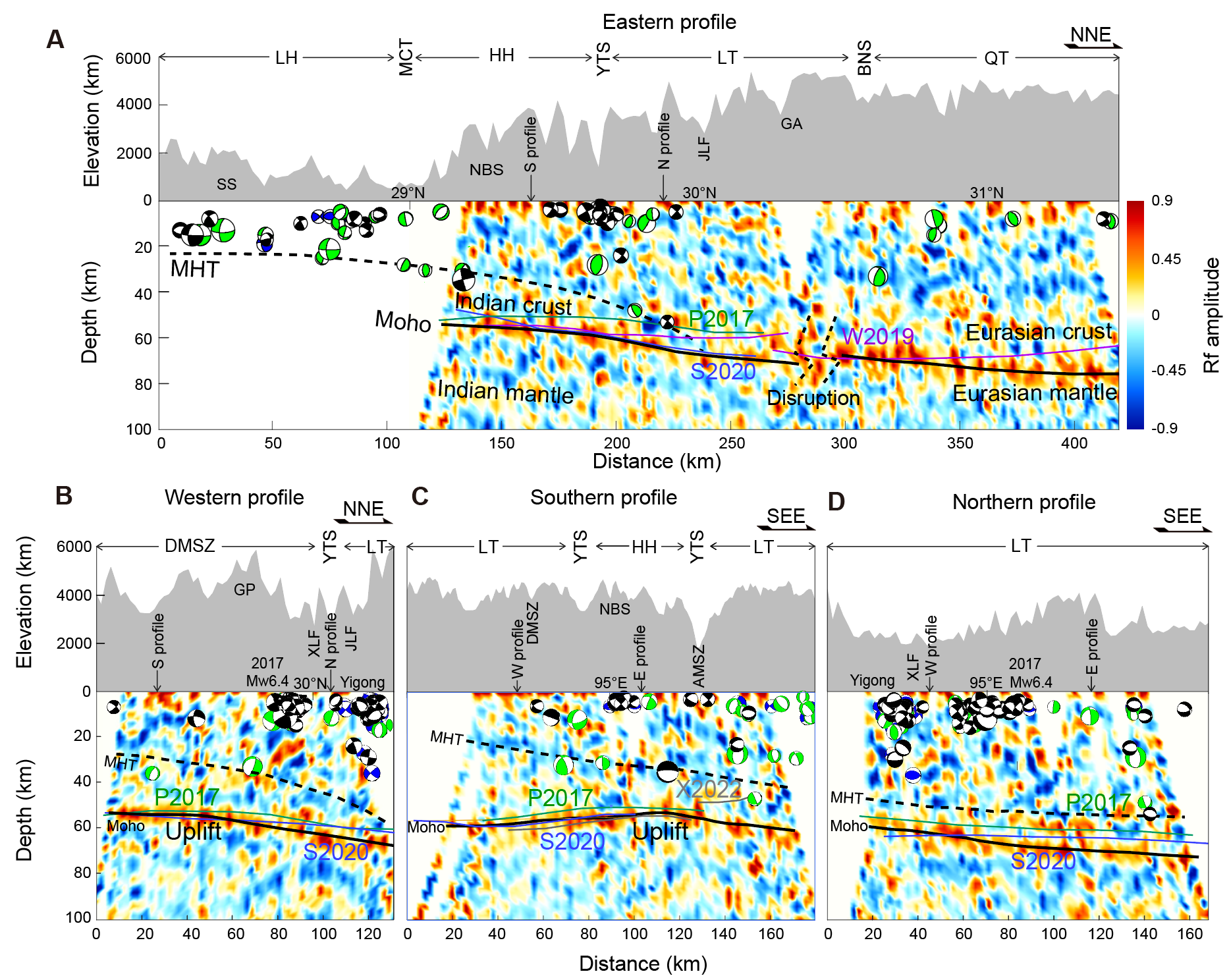


**Fig. S7.** Receiver function images with earthquakes for four profiles showing comparison of the Moho with previous studies. (A) The eastern profile at [(94.40°E 28.20°N), (96.50°E 31.50°N)]. (B) The western profile at [(94.46°E 29.30°N), (95.20°E 30.35°N)]. (C) The southern profile at [(94.10°E 29.68°N), (95.89°E 29.30°N)]. (D) the northern profile at [(94.50°E 30.38°N), (96.00°E 29.73°N)]. The black solid lines are the inferred Moho and the dotted lines are the inferred MHT. Other solid lines show constraints on Moho from receiver functions: P2017 [69], W2019 [22], and X2022 [50], and gravimetric observations: S2020 [70].

Table S1. List of seismic stations we deployed at the Eastern Himalayas.

| No. | Name | Longitude/deg | Latitude/deg | Altitude/m |
| --- | --- | --- | --- | --- |
| 1  2  3  4  5  6  7  8  9  10  11  12  13  14  15  16  17  18  19  20  21  22  23  24  25  26  27  28  29  30  31  32  33  34  35  36  37  38  39  40  41  42  43  44  45  46  47  48  49  50  51  52  53  54  55  56  57  58  59  60  61  62  63  64  65  66  67  68  69  70  71  72  73  74  75  76  77  78  79  80  81  82  83  84  85  86  87  88  89  90 | LZ00  LZ01  LZ04  LZ05  LZ06  LZ07  LZ08  LZ09  LZ10  LZ11  LZ12  LZ13  LZ15  LZ16  LZ17  LZ18  LZ19  LZ20  LZ21  LZ22  LZ23  ML01  ML02  ML03  ML04  ML05  ML06  ML07  ML08  ML09  ML10  ML11  ML12  ML13  ML14  ML15  ML16  ML17  ML18  ML19  ML20  ML21  ML22  BM01  BM02  BM03  BM04  BM05  BM06  BM07  BM08  BM09  MT00  MT01  MT02  MT03  MT04  MT05  MT06  MT07  MT08  MT09  MT10  MT11  CD01  CD02  CD03  CD04  CD05  CD06  CD07  CD08  CD09  CD10  CD11  CD12  CD13  CD14  CD15  CD16  CD17  CY01  CY02  CY03  CY04  CY05  CY06  CY07  CY08  CY09 | 94.7373  95.3497  95.3041  95.6113  95.4068  95.4901  95.4667  95.5817  95.7409  95.4806  95.2947  95.0872  94.8251  94.6429  94.7700  94.9043  94.9200  94.7043  94.5313  94.2590  94.7006  94.8687  94.7375  94.8296  95.0077  94.7546  95.2037  94.6830  95.4722  95.4510  95.1324  94.9038  94.6102  94.5312  94.4939  94.5310  95.3208  94.9393  94.3929  94.4338  94.7703  94.8162  94.9706  95.5930  95.6278  95.4666  95.6405  95.7173  95.7573  95.7667  95.7691  95.7882  95.0024  95.1284  95.1563  95.2287  95.3384  95.4077  95.4603  95.4932  95.6631  95.7366  95.5988  95.6947  97.1816  97.0818  96.9996  96.8456  96.7436  96.6433  96.5617  96.4708  96.3667  96.2873  96.2464  96.1765  96.0454  95.9074  95.7745  95.8469  95.7803  97.0507  96.7714  96.4861  96.7916  97.2188  97.4892  97.4623  97.3627  97.1896 | 29.7651  30.2612  29.3282  30.0025  29.4175  29.6599  29.5833  29.7103  29.8659  29.9130  30.0181  30.1030  30.2455  30.3110  29.9734  29.7052  29.5735  29.4449  29.4261  29.2507  29.9952  29.5134  29.7650  29.5133  30.0440  29.4883  30.0602  29.5219  29.9089  29.4745  29.2223  29.7014  29.5139  29.4265  29.5753  29.5526  29.3161  29.6180  29.6268  29.5986  29.9740  30.2759  30.1699  29.8778  29.9387  29.9994  30.0713  30.1095  30.1515  30.1969  30.2598  29.8230  29.4678  29.3671  29.2667  29.2929  29.3681  29.4197  29.5482  29.6667  29.4415  29.4664  29.4430  29.4542  31.1513  31.1832  31.0975  31.1116  31.1296  31.1517  31.2419  31.1601  31.0968  31.0199  30.9168  30.8136  30.7277  30.7493  30.6249  30.6694  30.5180  28.4121  28.7452  29.1012  29.4976  29.1469  28.8480  28.6664  28.6217  28.6022 | 3333  3031  816  2735  820  2098  1404  2770  2723  2765  2469  2077  2209  2536  2624  2828  3181  2933  2959  2943  2770  2980  3327  3016  2062  3019  2420  3457  2671  1433  834  2835  3103  2969  3164  3407  1231  2987  3040  3005  2642  2245  2180  2728  2744  2760  2784  2873  2982  3053  3233  2755  3233  2133  1464  1699  1291  843  1216  2166  1945  2161  1748  2083  3223  3296  3907  3856  3724  3731  3853  4005  4289  4200  4131  4151  4052  4037  4052  3911  4221  1460  1933  2234  4100  3249  2516  2359  2176  1856 |

**Table S2.** Source parameters of 164 earthquakes obtained in this study. ID is the number of earthquakes in origin time order. The date and origin time are GMT time. M_W_ is moment magnitude. Strike, dip, and slip are the three parameters of focal mechanisms.

| ID | year mm dd | hh mm ss.sss | longitude  /deg | latitude  /deg | depth  /km | Mw | strike dip slip |
| --- | --- | --- | --- | --- | --- | --- | --- |
| 1  2  3  4  5  6  7  8  9  10  11  12  13  14  15  16  17  18  19  20  21  22  23  24  25  26  27  28  29  30  31  32  33  34  35  36  37  38  39  40  41  42  43  44  45  46  47  48  49  50  51  52  53  54  55  56  57  58  59  60  61  62  63  64  65  66  67  68  69  70  71  72  73  74  75  76  77  78  79  80  81  82  83  84  85  86  87  88  89  90  91  92  93  94  95  96  97  98  99  100  101  102  103  104  105  106  107  108  109  110  111  112  113  114  115  116  117  118  119  120  121  122  123  124  125  126  127  128  129  130  131  132  133  134  135  136  137  138  139  140  141  142  143  144  145  146  147  148  149  150  151  152  153  154  155  156  157  158  159  160  161  162  163  164 | 2003 08 18  2003 08 18  2003 10 14  2003 12 03  2003 12 08  2004 02 08  2004 04 20  2004 04 20  2004 06 01  2004 09 11  2004 09 27  2004 10 17  2015 10 15  2015 10 24  2015 12 05  2015 12 10  2015 12 11  2015 12 16  2016 01 02  2016 02 21  2016 03 22  2017 11 23  2017 11 23  2017 11 23  2017 11 23  2017 11 24  2017 11 24  2017 11 25  2017 12 13  2017 12 19  2017 12 19  2017 12 19  2017 12 23  2017 12 31  2018 01 10  2018 02 08  2018 02 09  2018 02 25  2018 03 13  2018 03 15  2018 03 25  2018 04 03  2018 06 02  2018 07 16  2018 07 26  2018 07 27  2018 08 18  2018 08 22  2018 10 26  2018 10 30  2018 11 07  2018 11 08  2018 11 09  2018 11 22  2018 11 22  2018 11 28  2018 11 29  2019 01 12  2019 01 12  2019 01 17  2019 01 20  2019 02 10  2019 02 22  2019 03 15  2019 04 04  2019 04 23  2019 05 02  2019 05 07  2019 05 09  2019 06 14  2019 07 23  2019 08 20  2019 09 01  2019 09 08  2019 09 23  2019 10 03  2019 10 09  2019 10 24  2019 11 01  2020 03 15  2020 04 29  2020 04 29  2020 05 01  2020 05 12  2020 05 20  2020 07 06  2020 07 19  2020 07 19  2020 07 20  2020 07 20  2020 07 21  2020 07 21  2020 07 21  2020 07 23  2020 07 23  2020 07 24  2020 07 25  2020 07 26  2020 07 27  2020 07 28  2020 07 29  2020 07 29  2020 07 30  2020 07 31  2020 07 31  2020 08 02  2020 08 08  2020 08 09  2020 08 09  2020 08 11  2020 08 11  2020 08 12  2020 08 13  2020 08 13  2020 08 14  2020 08 16  2020 08 17  2020 11 08  2020 11 16  2020 11 18  2020 11 22  2020 11 22  2020 11 22  2020 11 28  2020 11 29  2020 12 02  2020 12 08  2020 12 22  2020 12 22  2020 12 23  2021 01 24  2021 02 05  2021 02 07  2021 02 09  2021 03 05  2021 03 23  2021 04 13  2021 04 17  2021 05 01  2021 09 19  2021 09 25  2021 10 01  2021 10 01  2021 10 02  2021 10 04  2021 10 05  2021 10 05  2021 10 09  2021 10 10  2021 10 10  2021 10 12  2021 10 12  2021 11 28  2021 12 14  2021 12 21  2021 12 21  2022 01 17  2022 01 21  2022 01 25  2022 01 28  2022 02 06  2022 02 20  2022 03 03  2022 03 15 | 09 03 02.072  09 37 18.777  22 56 58.883  19 47 46.391  20 41 36.531  22 10 29.938  08 17 52.711  21 56 39.977  05 05 48.684  17 40 43.215  17 05 36.789  01 31 29.395  16 10 49.000  19 55 32.398  03 23 10.691  23 50 48.727  22 09 35.984  10 25 19.859  18 27 34.961  07 06 33.000  14 54 50.609  02 14 12.225  07 13 44.973  14 12 37.984  19 26 31.898  01 58 03.479  10 51 33.676  02 51 07.993  10 17 30.734  17 40 51.723  17 43 11.965  20 08 42.422  14 06 16.191  05 47 47.123  22 35 32.156  19 48 40.555  11 30 41.398  17 16 14.078  13 10 09.461  03 40 13.309  12 38 55.242  21 33 40.656  05 45 58.000  09 47 43.836  05 17 16.184  19 52 13.148  20 29 41.000  20 46 47.000  04 20 44.385  12 56 57.906  21 55 58.453  07 37 43.754  22 55 15.102  16 53 11.375  18 59 19.500  10 17 05.117  23 55 20.984  09 43 39.938  20 13 47.922  06 04 47.391  02 52 01.229  03 22 03.000  19 36 35.359  16 30 19.629  12 58 28.219  20 39 35.938  08 10 21.586  03 42 02.027  03 54 10.251  04 12 42.979  06 56 58.029  01 08 50.846  17 19 44.973  00 08 41.492  09 36 28.676  17 26 54.000  23 14 28.031  09 43 29.676  04 05 52.277  13 10 46.539  18 41 52.281  21 51 31.844  17 30 09.918  01 53 55.535  17 32 08.465  20 23 31.516  10 15 43.891  23 36 35.000  19 21 37.117  19 39 14.305  02 15 16.936  06 18 46.006  13 26 14.539  00 47 41.607  09 25 09.984  13 34 21.855  15 58 34.961  09 58 54.871  05 50 17.170  03 32 46.278  11 07 08.969  17 27 50.418  18 38 35.891  16 45 08.504  18 58 47.000  17 27 37.940  08 43 57.080  08 50 10.844  17 13 30.297  18 14 13.680  21 27 25.680  15 58 06.316  04 27 56.526  22 11 32.391  12 58 38.570  16 40 37.391  16 31 49.203  09 12 24.629  01 39 04.796  05 51 11.477  03 29 50.806  15 19 57.406  16 37 37.668  23 40 41.609  06 18 11.180  07 13 52.881  16 45 10.031  20 04 39.281  20 08 39.516  11 06 42.801  07 06 10.006  19 05 51.555  13 02 10.184  09 47 24.801  01 26 02.285  22 43 41.352  15 26 08.266  05 40 17.932  11 53 21.473  09 36 29.500  04 41 01.600  03 49 22.415  13 01 54.875  04 45 18.869  03 24 16.362  02 38 15.131  23 27 51.758  05 37 57.863  01 48 49.463  16 29 36.305  00 31 50.121  20 24 13.539  17 02 21.000  12 32 15.348  05 04 14.000  22 36 58.602  22 59 29.313  15 18 04.297  02 51 13.392  12 35 04.715  17 29 11.582  23 09 48.023  08 47 04.354  02 51 00.444 | 95.6229  95.6008  95.7964  95.1679  95.7777  96.8516  94.3283  94.1755  95.6467  95.4571  95.5406  97.1606  95.2200  96.1554  95.7598  96.6376  95.6790  95.3760  94.8356  96.1400  96.0956  95.1583  95.1413  95.0600  94.8797  95.0723  95.0625  94.9349  95.8172  95.0523  95.0580  95.0370  95.2039  95.1595  95.1624  95.8243  95.1709  95.0650  94.7616  94.7483  95.1336  96.0053  96.8900  95.0982  95.0170  98.0559  95.7900  95.7900  96.8296  94.9334  94.9277  95.7459  95.3189  94.9324  94.9989  96.9702  94.9524  95.8940  94.1985  94.8482  94.9376  96.3700  97.0764  95.2867  93.8471  94.5776  96.4393  94.2018  95.0679  93.2857  96.0868  94.8622  95.8007  95.4090  94.9139  95.4600  96.3948  96.2494  95.0608  95.2721  94.8369  95.6862  94.6823  96.6412  95.4117  96.0147  94.8420  94.9190  94.8332  95.6822  94.8540  94.8434  94.8496  94.8722  95.7495  94.8445  94.8508  94.8492  94.8523  94.8705  94.8244  94.8053  94.9419  94.7239  94.8700  95.6580  94.8811  94.8790  94.8619  94.8968  94.8746  95.0940  94.8891  95.1471  94.8608  94.8512  94.8961  96.4165  93.3943  96.8805  95.0152  96.7039  97.3628  94.9014  94.8935  95.7372  95.0670  94.9549  95.0189  96.7990  94.9834  95.6088  96.0762  95.8575  95.7963  95.9912  96.9542  94.7747  95.5330  96.3300  94.8306  94.8394  94.8211  94.8568  94.8425  94.8574  94.8543  94.8811  94.8231  94.8313  94.8296  94.9144  96.3200  95.8033  96.9900  95.6685  94.1855  95.6126  95.0102  96.8810  96.2376  96.2223  94.9608  94.9079 | 29.5702  29.5503  27.2445  29.8151  29.8483  28.7713  28.8575  28.9505  29.6637  29.4835  29.8333  29.4859  28.5000  28.1007  28.9936  28.0767  29.6844  29.4804  28.4697  28.5500  28.2869  29.7961  29.8624  29.8948  30.1844  29.9017  29.8986  29.9859  29.0657  29.9244  29.9246  29.9264  29.8089  29.8129  29.8358  29.7850  29.8811  29.8854  28.9480  29.0033  28.2757  28.0550  27.0600  29.8515  28.6993  28.7612  28.7400  28.8300  29.4195  29.9403  29.9185  28.5027  28.4836  29.9526  29.9155  27.2130  29.9387  29.0683  28.6685  28.6161  29.9461  27.3700  29.1135  28.9557  28.1260  28.3407  28.3250  28.3942  29.8954  27.7623  28.7970  30.3512  29.0642  29.9268  29.3075  28.1200  28.6698  28.5565  29.8642  28.8787  28.9185  28.6849  28.8965  30.9405  28.9386  28.7820  30.4087  30.3328  30.3728  29.5857  30.3847  30.4009  30.3745  30.3803  29.3005  30.3731  30.3557  30.3739  30.4050  30.3825  30.4073  30.4275  30.3592  30.2492  30.3700  28.5740  30.3815  30.3607  30.3746  30.3857  30.3656  29.8459  30.3583  29.8339  30.3616  30.3743  30.3613  28.6214  31.4531  30.0493  28.6293  29.3542  30.7621  29.9267  29.9186  29.6811  31.9937  31.8985  31.8983  26.9907  29.9399  28.6951  28.0504  29.0620  29.2220  28.8013  27.3825  30.2431  29.0888  28.5600  30.3905  30.3961  30.4143  30.3729  30.4004  30.3544  30.4109  30.3747  30.4036  30.4168  30.3709  30.4201  27.0400  28.9859  27.5600  29.1404  29.1763  28.6076  28.9999  30.2778  30.8088  30.7932  29.9058  29.9316 | 28.0  7.0  5.0  5.0  5.0  6.0  25.0  30.0  24.0  4.0  10.0  7.0  15.0  10.0  3.0  18.0  9.0  4.0  17.0  18.0  14.0  4.0  8.0  9.0  6.0  7.0  7.0  5.0  12.0  6.0  6.0  2.0  5.0  3.0  7.0  53.0  6.0  4.0  10.0  7.0  5.0  10.0  9.0  6.0  10.0  8.0  14.0  17.0  4.0  10.0  7.0  9.0  9.0  13.0  7.0  10.0  11.0  15.0  9.0  10.0  14.0  4.0  7.0  32.0  6.0  8.0  14.0  13.0  6.0  11.0  9.0  6.0  8.0  6.0  32.0  14.0  6.0  13.0  8.0  8.0  13.0  9.0  14.0  8.0  16.0  10.0  6.0  36.0  6.0  10.0  8.0  7.0  8.0  8.0  6.0  9.0  6.0  6.0  9.0  6.0  6.0  6.0  10.0  12.0  7.0  10.0  6.0  5.0  5.0  8.0  8.0  3.0  5.0  4.0  6.0  6.0  6.0  15.0  25.0  10.0  25.0  14.0  10.0  6.0  4.0  48.0  7.0  4.0  9.0  11.0  15.0  5.0  5.0  17.0  30.0  8.0  10.0  4.0  11.0  7.0  5.0  7.0  5.0  5.0  8.0  6.0  6.0  5.0  5.0  5.0  6.0  15.0  7.0  17.0  8.0  29.0  30.0  15.0  28.0  17.0  11.0  15.0  11.0  8.0 | 5.53  4.13  3.55  3.70  3.75  3.69  3.77  3.65  3.91  3.86  4.83  3.94  3.63  3.78  3.27  3.40  3.39  3.85  3.39  3.32  3.51  4.19  4.42  3.75  3.35  3.31  3.75  3.74  3.60  5.19  3.97  3.90  3.26  3.36  3.46  3.61  3.51  3.69  3.41  3.43  3.66  3.57  4.40  3.15  3.59  3.86  3.63  3.78  4.00  4.03  3.66  3.98  3.82  3.54  3.82  5.01  3.79  3.52  3.69  3.64  4.29  4.93  3.75  3.41  3.81  4.18  3.77  4.00  3.62  3.88  4.36  3.71  3.98  3.38  3.78  4.02  3.92  3.76  3.61  3.79  3.66  3.58  3.46  4.08  3.68  4.07  4.45  4.22  4.19  3.66  3.57  4.11  3.75  4.17  4.18  4.28  4.27  4.20  4.28  4.26  4.35  4.04  4.22  4.13  3.79  3.73  4.28  4.28  4.07  4.20  3.90  4.36  4.03  3.58  4.06  4.11  4.25  4.13  4.12  3.67  3.65  3.66  3.97  3.26  3.78  3.70  3.83  3.76  3.37  3.35  3.72  3.60  3.52  3.80  3.75  3.55  3.19  3.39  3.36  4.17  4.09  4.06  4.01  3.89  3.88  3.86  3.66  3.75  3.71  3.96  3.39  3.21  3.70  3.67  4.07  3.41  4.49  3.62  3.61  3.61  3.97  3.69  3.77  3.85 | 155 80 -173  234 79 15  109 41 81  345 44 87  315 45 98  347 84 163  50 64 110  131 49 89  136 51 91  214 45 101  36 85 -29  133 90 170  335 84 66  139 90 105  143 90 169  98 86 175  166 85 -154  346 35 115  350 86 101  14 87 1  287 81 170  268 89 97  136 25 89  45 33 95  321 45 81  286 39 80  259 73 -33  251 40 108  292 75 168  101 54 89  125 73 -156  160 89 85  319 66 67  354 55 105  254 44 76  314 39 106  261 76 117  156 58 86  170 49 109  240 69 176  316 89 -180  295 83 -169  240 35 114  320 45 69  318 86 -166  343 82 -180  269 65 171  286 73 171  264 55 106  165 49 123  220 59 76  280 73 -176  25 79 26  55 49 95  60 45 108  171 31 85  195 70 27  99 81 -171  20 37 88  311 60 124  171 64 -165  165 11 90  141 65 -156  174 87 177  100 54 54  83 51 55  5 90 -4  35 48 90  80 55 90  96 90 -176  15 85 4  65 43 80  137 83 -135  201 84 -4  9 89 -26  124 90 173  163 67 -148  96 87 180  80 41 100  294 80 168  155 54 95  117 84 171  120 74 149  253 68 -20  161 70 -143  282 86 -180  105 44 -89  105 49 -91  278 45 -99  91 35 85  104 45 -70  56 49 -118  79 46 -85  284 45 -86  344 50 165  105 48 -80  275 42 -89  55 50 -121  271 44 84  115 43 -95  282 44 -82  285 46 -71  240 50 -116  220 71 21  80 44 -86  95 80 171  110 48 -81  293 43 -82  276 43 -104  278 45 -104  230 51 -116  85 26 93  114 43 -85  64 45 70  277 44 -87  284 44 -79  271 47 86  150 71 -166  89 80 -161  105 84 180  136 79 167  156 79 173  190 77 174  223 52 -39  355 87 180  58 82 17  284 76 10  136 45 90  347 43 87  289 57 149  69 39 111  216 80 32  285 67 166  148 74 -160  133 64 -153  293 75 173  0 29 129  173 65 46  341 51 -153  160 62 -163  296 44 84  260 48 87  295 46 85  296 44 84  308 45 71  114 82 -158  305 43 -85  123 48 -87  310 50 78  115 39 91  149 50 103  157 90 172  225 51 56  295 56 180  340 30 100  254 80 -13  234 60 5  303 69 173  196 84 -155  140 83 161  115 89 175  144 76 -175  15 66 10  214 45 85 |
